# Supplementary material for: A transmission-virulence evolutionary trade-off explains attenuation of HIV-1 in Uganda
Source: eLife. 2016 Nov 5;5:e20492. doi: 10.7554/eLife.20492 (PMC5115872; doi:10.7554/eLife.20492)
Supplement: Figure 1—source data 1. — (A) Model comparison for the transmission rate as a function of SPVL and other covariates, based on the Akaike Information Criterion. d.f. are the degrees of freedom, N is the sample size.(B) Model comparison for the time to AIDS as a function of SPVL and other covariates, based on the Akaike Information Criterion. d.f. are the degrees of freedom, N is the sample size. DOI: http://dx.doi.org/10.7554/eLife.20492.005 [file elife-20492-fig1-data1.docx]

**Figure 1–source data 1. Data file for figure 1.**

| **Model** | **d.f.** | **N** | **AIC** | **ΔAIC** |
| --- | --- | --- | --- | --- |
| Flat (null model) | 1 | 817 | 1473.47 | 79.28 |
| Power | 2 | 817 | 1403.1 | 8.91 |
| Hill | 3 | 817 | 1399.14 | 4.95 |
| Hill-generalised | 5 | 817 | 1402.3 | 8.11 |
| **3 steps** | **5** | **817** | **1397.51** | **3.32** |
| 4 steps | 7 | 817 | 1402.45 | 8.26 |
| **3 steps - subtype** | **15** | **817** | **1394.19** | **0** |
| 3 steps - gender | 10 | 817 | 1399.17 | 4.98 |
| 3 steps - male index | 5 | 487 | 921.28 | 3.17 |
| **3 steps** **- male index - circumcision** | 10 | 487 | 918.11 | 0 |
| 3 steps - female index | 5 | 321 | 460.41 | 3.74 |
| **3 steps - female index - circumcision** | 10 | 321 | 456.67 | 0 |

**Figure 1–source data 1. Data file for figure 1. (A)** Model comparison for the transmission rate as a function of SPVL and other covariates, based on the Akaike Information Criterion. d.f. are the degrees of freedom, N is the sample size.

| **Model** | **d.f.** | **N** | **AIC** | **ΔAIC** |
| --- | --- | --- | --- | --- |
| Flat (null model) | 2 | 562 | 1585.57 | 137.22 |
| Power | 3 | 562 | 1461.93 | 13.58 |
| Hill | 4 | 562 | 1473.64 | 25.29 |
| Hill-generalised | 6 | 562 | 1463.52 | 15.17 |
| **3 steps** | **6** | **562** | **1448.35** | **0** |
| 3 steps - subtype | 16 | 562 | 1463.76 | 15.41 |
| 3 steps - gender | 11 | 562 | 1456.2 | 7.85 |

**Figure 1–source data 1. Data file for figure 1. (B)** Model comparison for the time to AIDS as a function of SPVL and other covariates, based on the Akaike Information Criterion. d.f. are the degrees of freedom, N is the sample size.
