# Supplementary material for: A transmission-virulence evolutionary trade-off explains attenuation of HIV-1 in Uganda
Source: eLife. 2016 Nov 5;5:e20492. doi: 10.7554/eLife.20492 (PMC5115872; doi:10.7554/eLife.20492)
Supplement: Figure 2—source data 1. — Summary of adjusted effects for the linear model explaining SPVL as a function of epidemiological covariates and date of seroconversion. The linear models included all the covariates listed. Effects significant in the whole dataset are in bold ‘p<0.1, *p<0.05, **p<0.01, ***p<0.001. DOI: http://dx.doi.org/10.7554/eLife.20492.010 [file elife-20492-fig2-data1.docx]

| **Factor** | **All SPVL**  **(n = 603)** | **Strict SPVL**  **(n = 240)** | **Abbott**  **(n = 31)** | **Roche1.5**  **(n = 572)** | **WR**  **(n = 299)** | **JH**  **(n = 129)** | **RHSP**  **(n = 175)** | **Male**  **(n = 268)** | **Female**  **(n = 335)** | **Subtype A** **(n = 94)** | **Subtype D** **(n = 285)** | **Other /unknown subtype** **(n = 224)** |
| --- | --- | --- | --- | --- | --- | --- | --- | --- | --- | --- | --- | --- |
| John Hopkins | 0 | 0 | - | 0 | - | - | - | 0 | 0 | 0 | 0 | 0 |
| RHSP | 0.154 | 0.551 ** | - | 0.147 | - | - | - | 0.363 * | -0.072 | -0.266 | 0.204 | 0.208 |
| Walter Reed | 0 | 0.287 ' | - | 0 | - | - | - | 0.108 | -0.146 | 0.085 | 0.012 | -0.22 |
| Abbott | 0 | 0 | - | - | - | - | 0 | 0 | 0 | - | 0 | 0 |
| Roche 1.5 | -0.189 | 1.048 * | - | - | - | - | -0.136 | -0.081 | -0.101 | - | 0.094 | -0.534 * |
| Not RCCS visit | 0 | 0 | 0 | 0 | - | - | 0 | 0 | 0 | 0 | 0 | 0 |
| **RCCS visit** | **-0.37 **** | -0.268 | -0.112 | -0.368 ** | - | - | -0.444 ** | -0.49 ** | -0.132 | 0.271 | -0.264 | -0.738 *** |
| Female | 0 | 0 | 0 | 0 | 0 | 0 | 0 | - | - | 0 | 0 | 0 |
| **Male** | **0.265 ***** | 0.404 *** | -0.108 | 0.273 *** | 0.277 ** | 0.16 | 0.375 ** | - | - | 0.321 * | 0.148 | 0.378 ** |
| Circumcised | - | - | - | - | - | - | - | 0 | - | - | - | - |
| Not Circumcised | - | - | - | - | - | - | - | -0.036 | - | - | - | - |
| **Age** | **0.008 *** | -0.004 | 0 | 0.008 * | 0.01 ' | 0.011 | 0.004 | 0.014 * | 0.004 | 0.023 * | 0.012 * | -0.003 |
| **Date seroconversion** | **-0.033 **** | -0.039 ' | -0.096 ' | -0.032 * | 0 | -0.07 * | -0.027 | -0.04 * | -0.022 | -0.048 ' | -0.026 | -0.038 ' |
| Subtype A | 0 | 0 | - | 0 | 0 | 0 | 0 | 0 | 0 | - | - | - |
| Subtype C | -0.447 | 0.284 | - | -0.448 | -0.318 | -0.874 | -0.447 | -0.606 | -0.399 | - | - | - |
| **Subtype D** | **0.213 *** | 0.271 ' | 0 | 0.216 * | 0.184 ' | 0.121 | 0.371 ' | 0.103 | 0.296 * | - | - | - |
| Recombinant | 0.092 | -0.003 | 1.855 * | 0.078 | 0.094 | 0.291 | -0.024 | 0.153 | 0.038 | - | - | - |
| Dual infections | -0.236 | -0.214 | - | -0.233 | -0.267 | -1.844 * | 0.284 | -0.467 | -0.144 | - | - | - |

**Figure 2–source data 1. Data file for figure 2**. Summary of adjusted effects for the linear model explaining SPVL as a function of epidemiological covariates and date of seroconversion. The linear models included all the covariates listed. Effects significant in the whole dataset are in bold ‘ p < 0.1, * p < 0.05, ** p < 0.01, *** p<0.001.
