# Supplementary figures and images for: A transmission-virulence evolutionary trade-off explains attenuation of HIV-1 in Uganda
Source: eLife. 2016 Nov 5;5:e20492. doi: 10.7554/eLife.20492 (PMC5115872; doi:10.7554/eLife.20492)

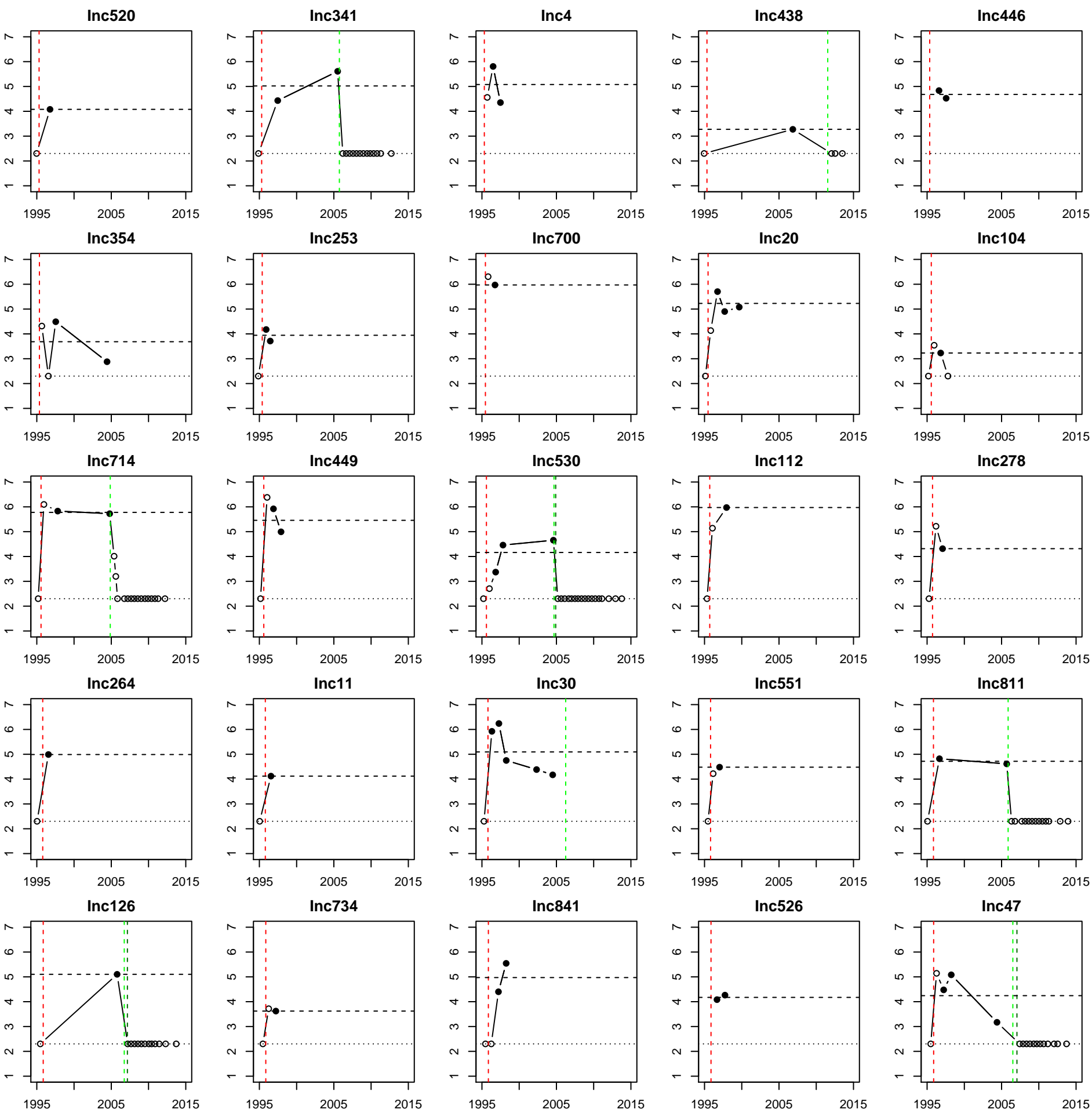

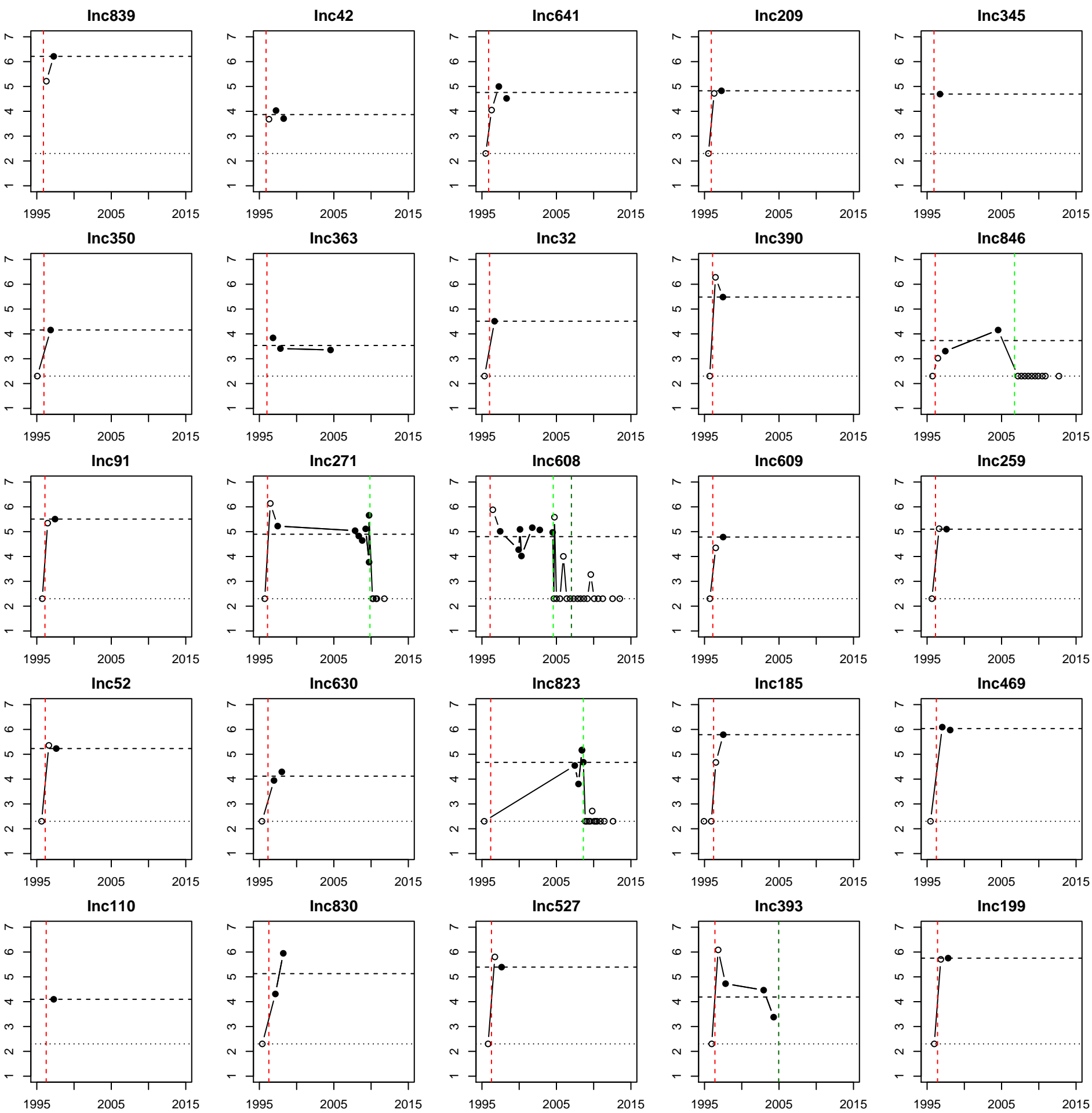

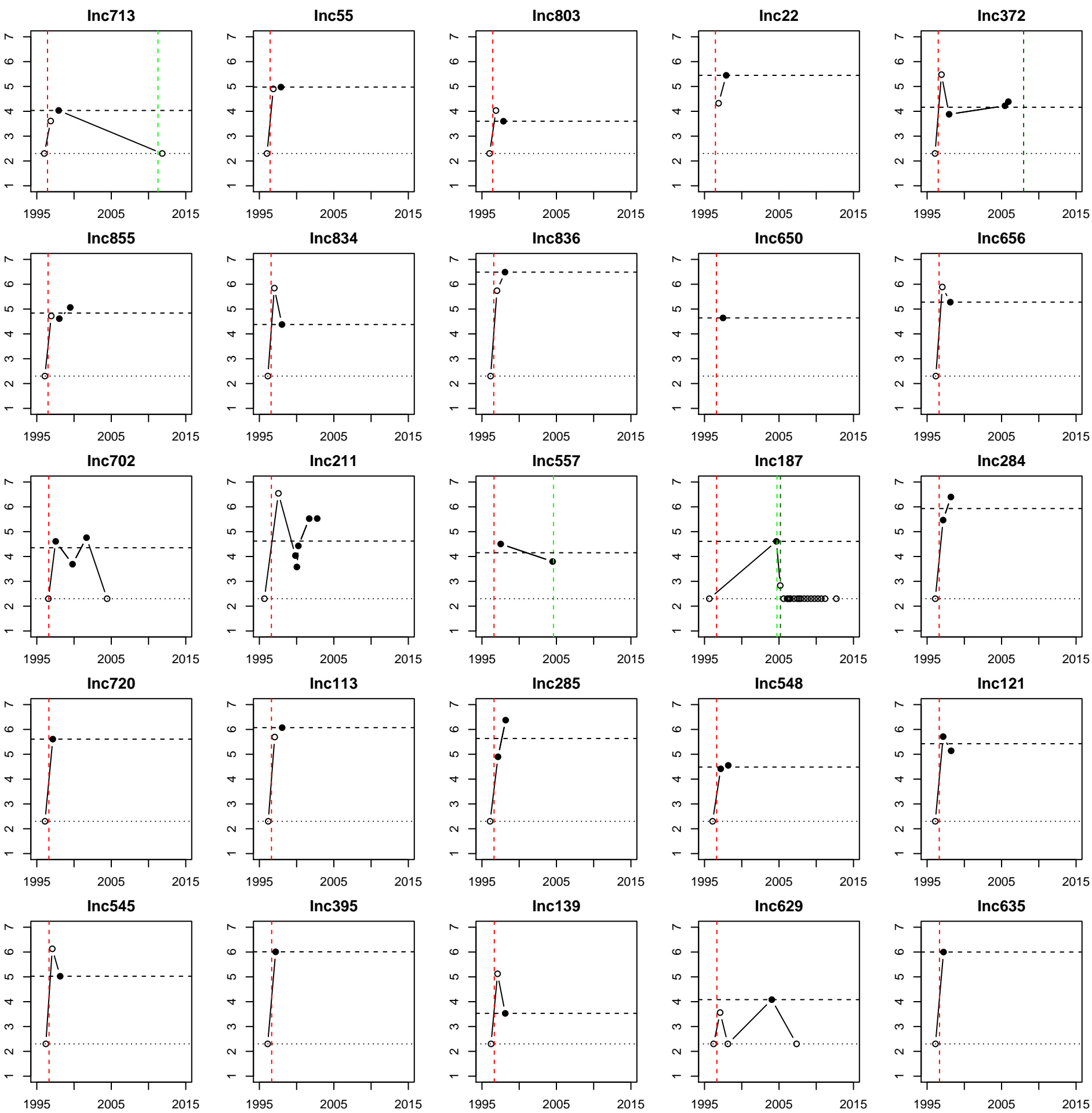

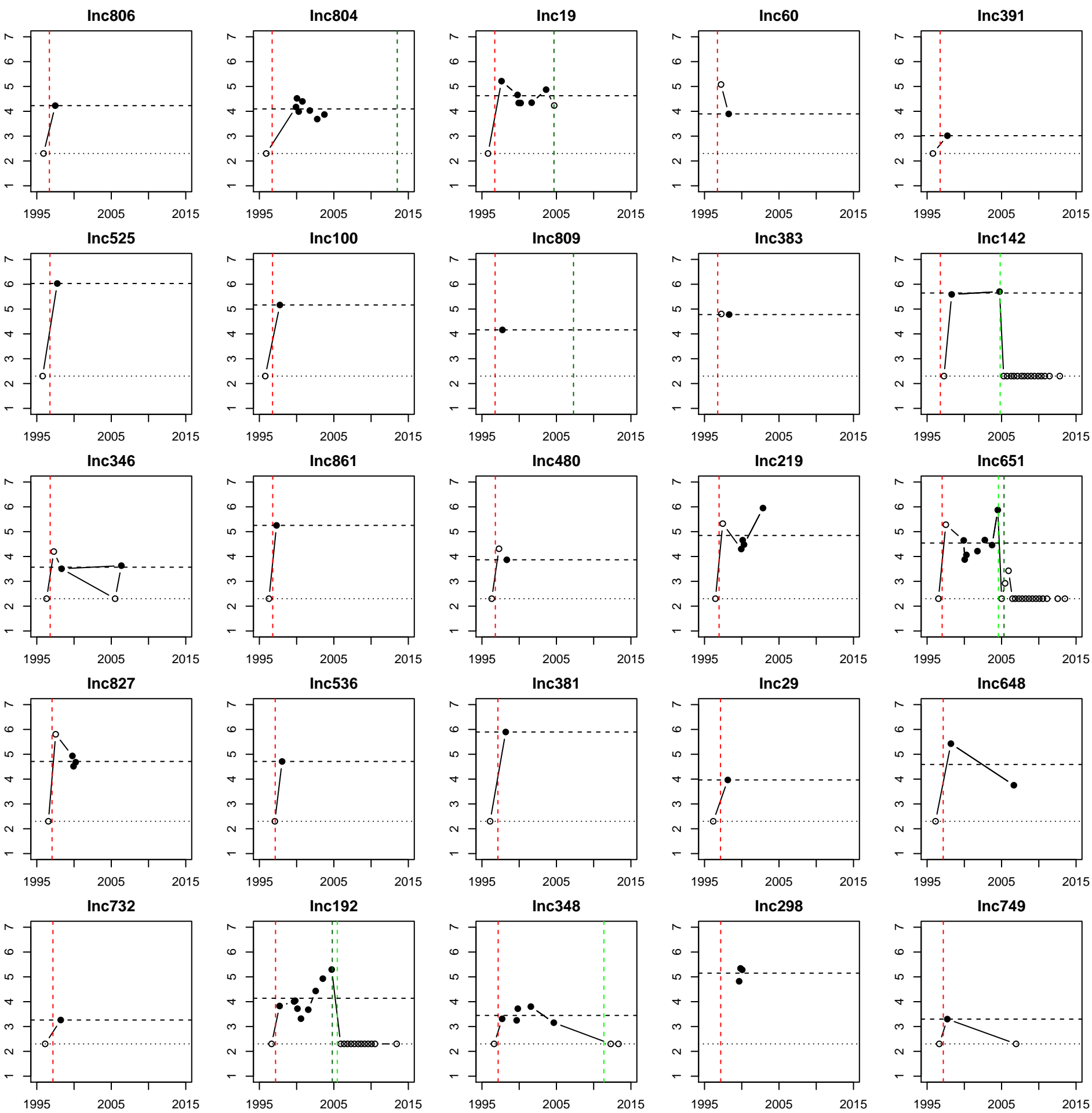

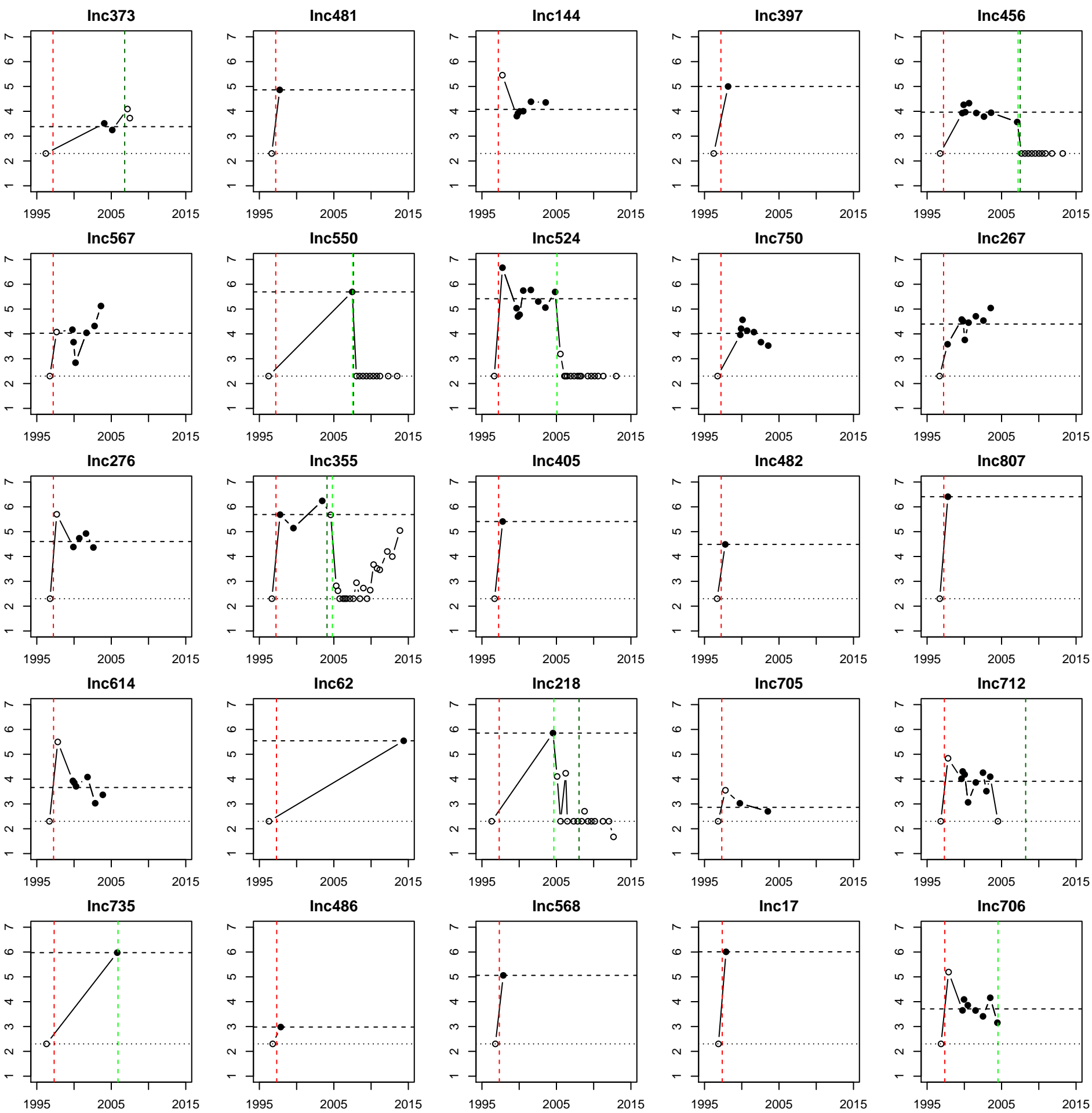

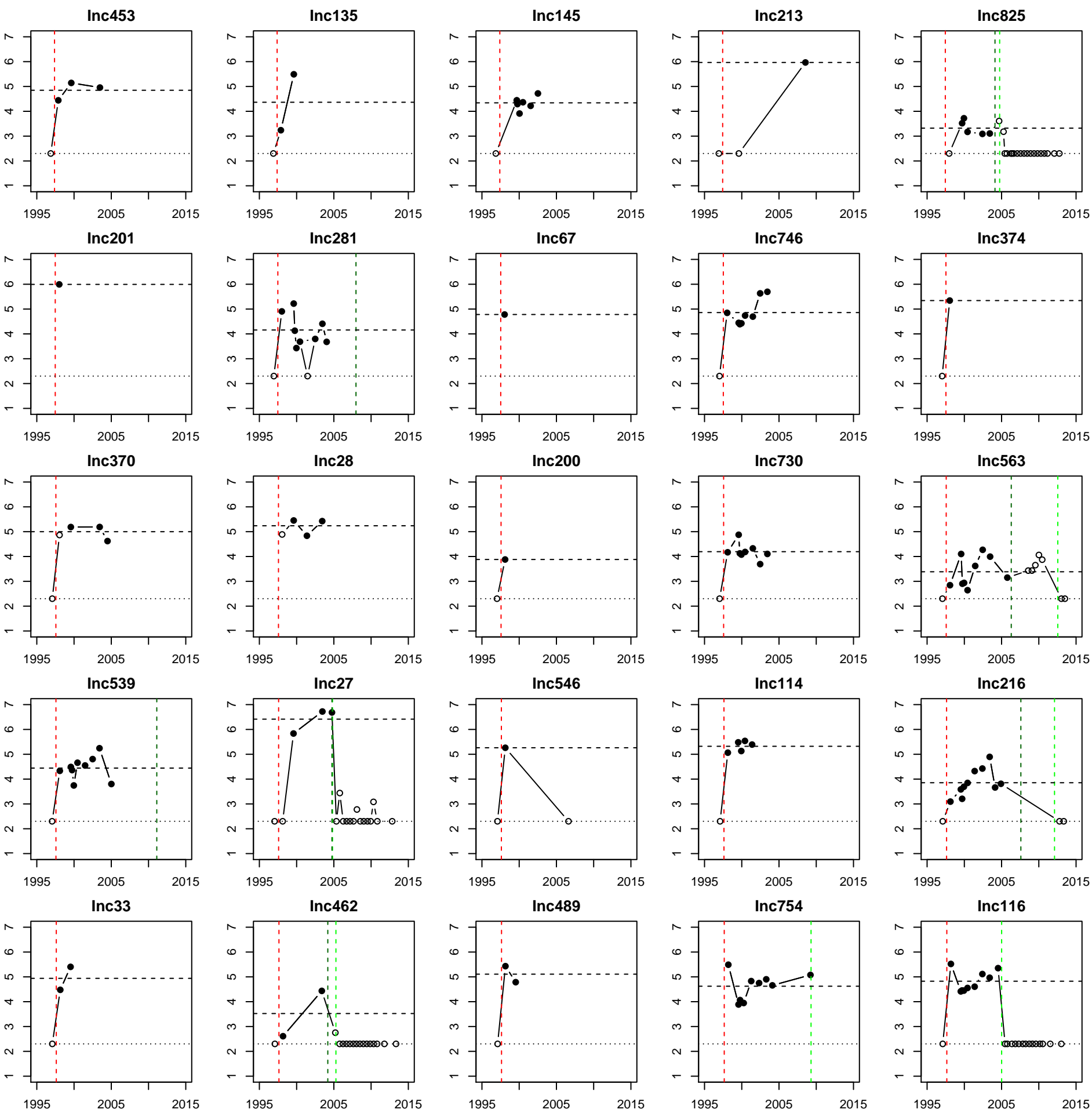

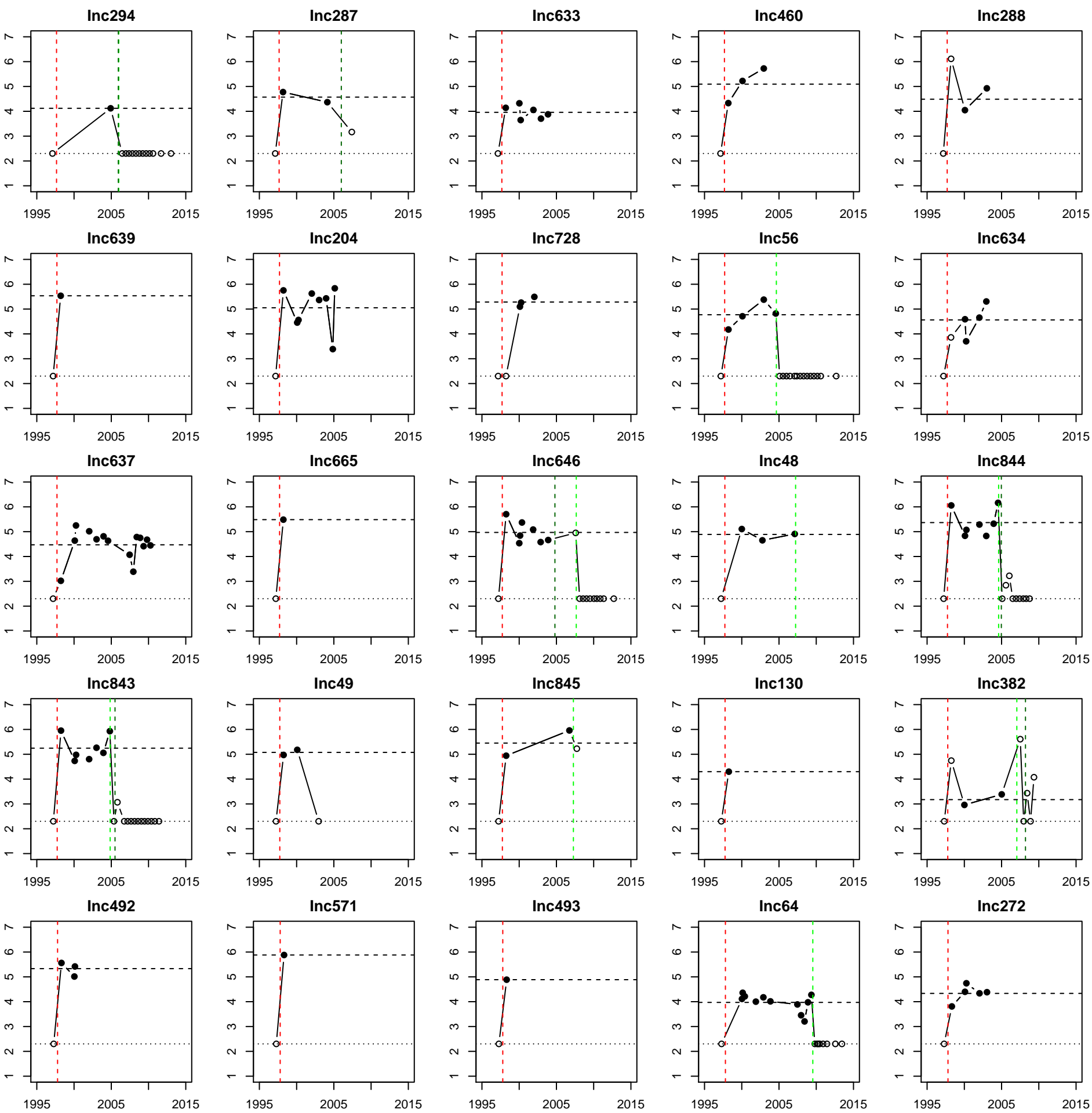

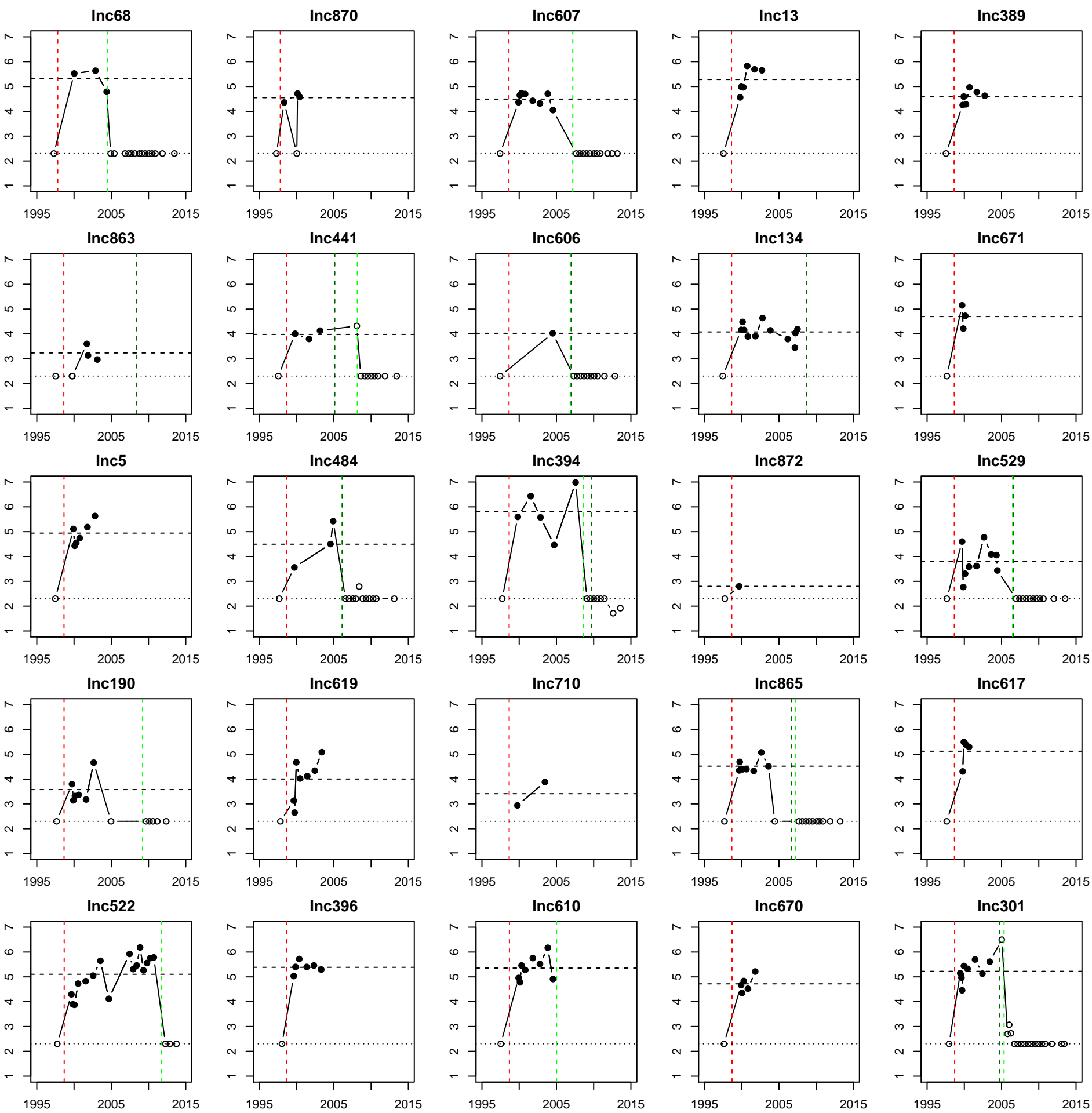

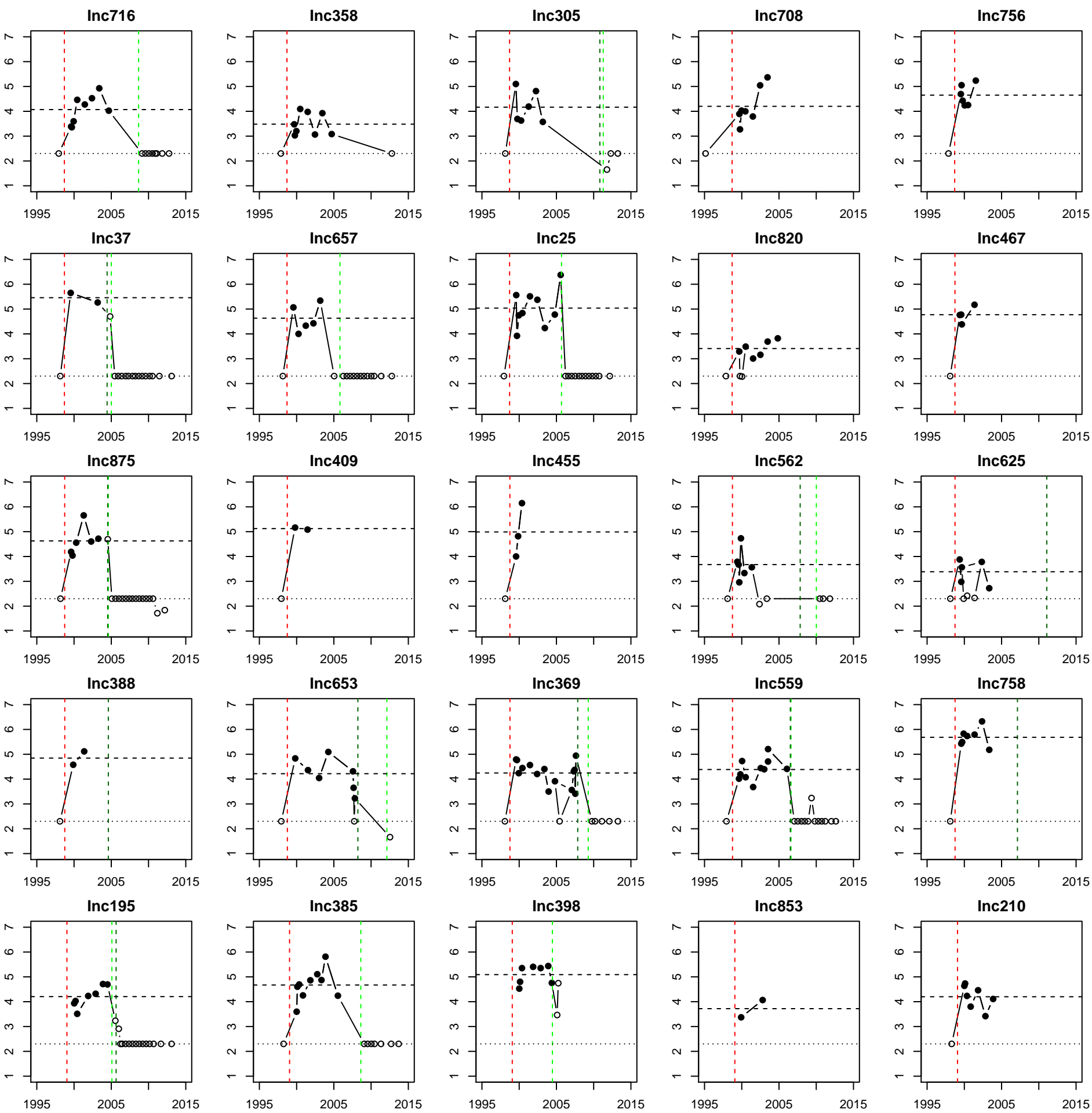

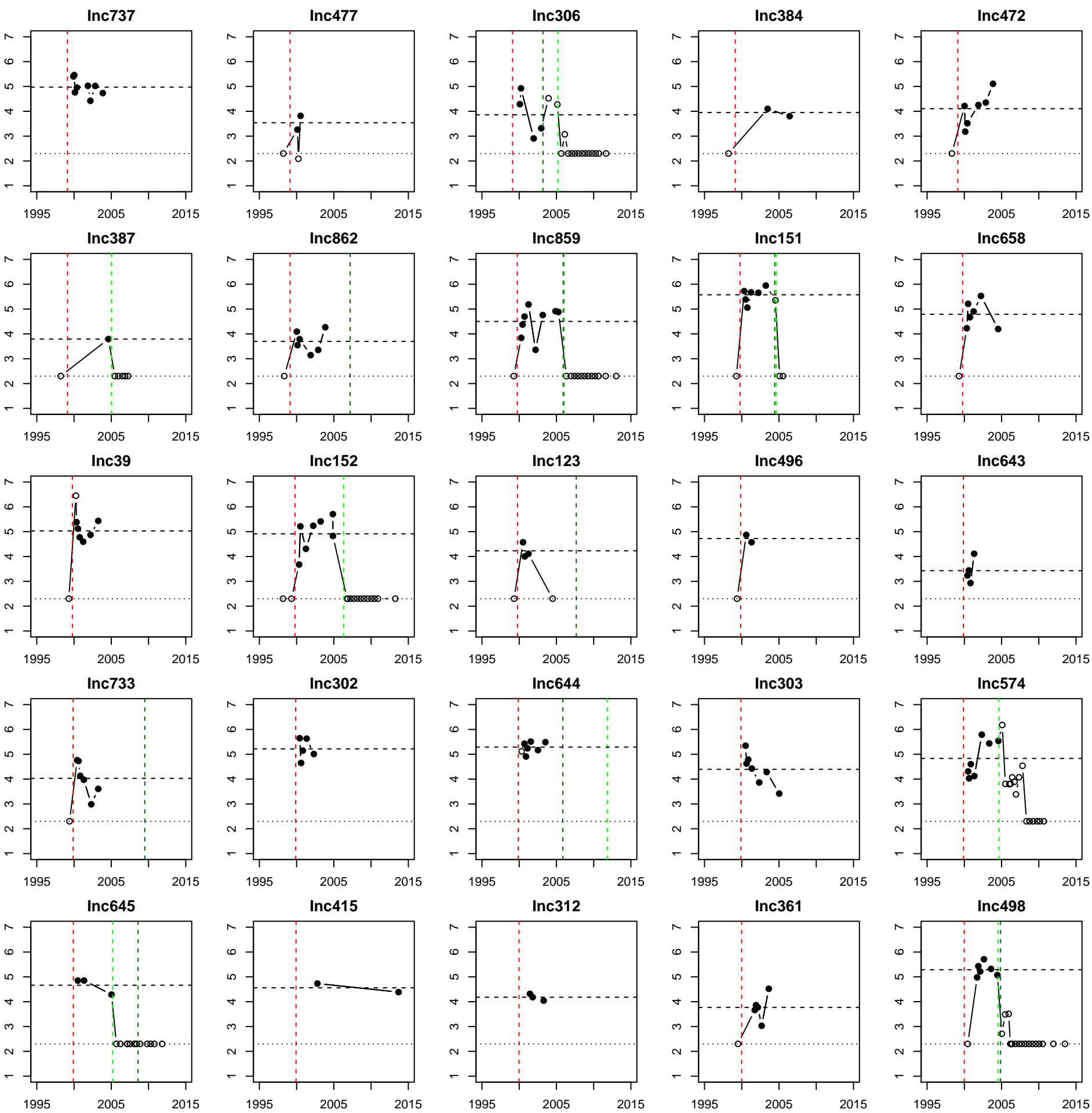

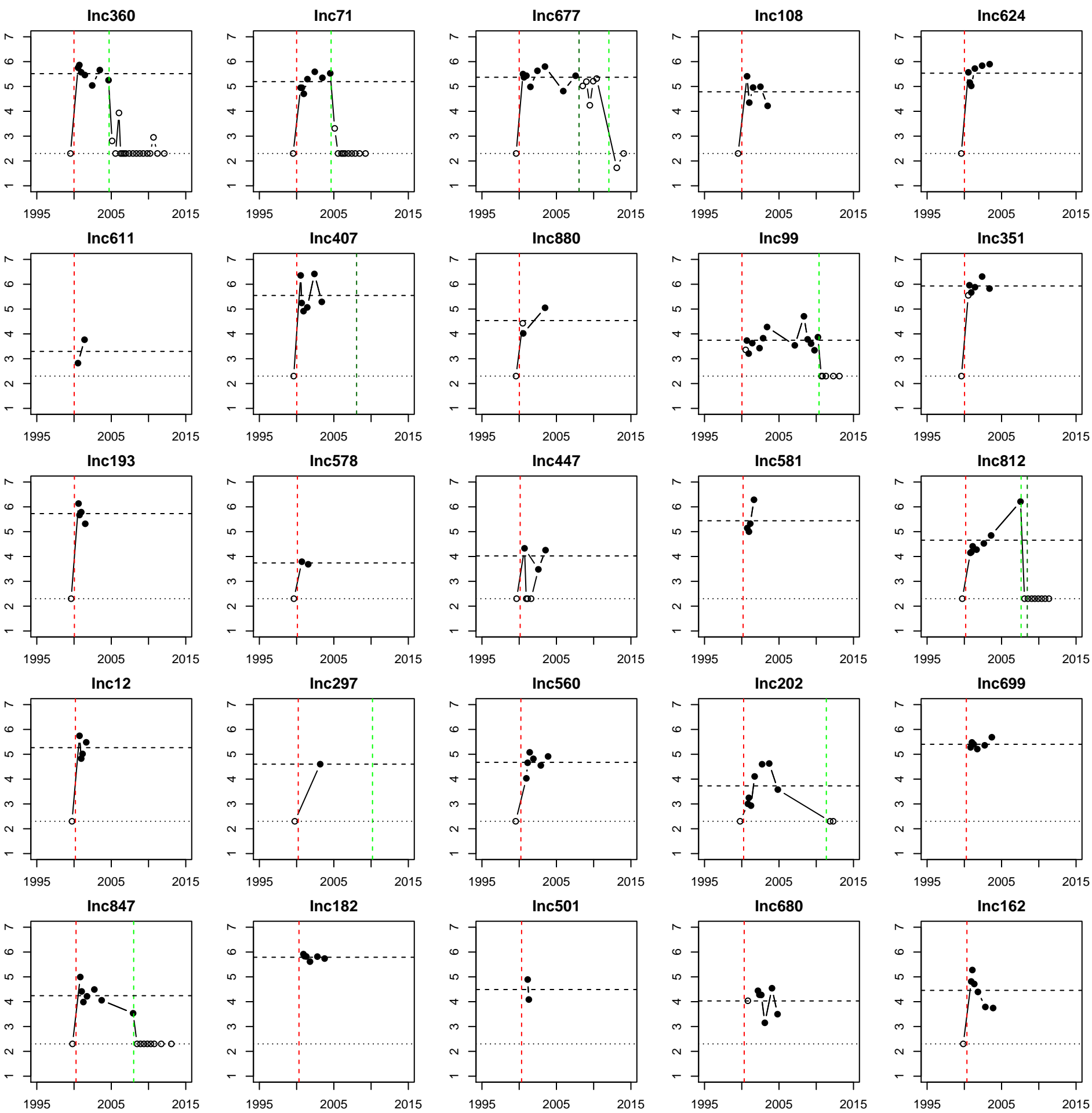

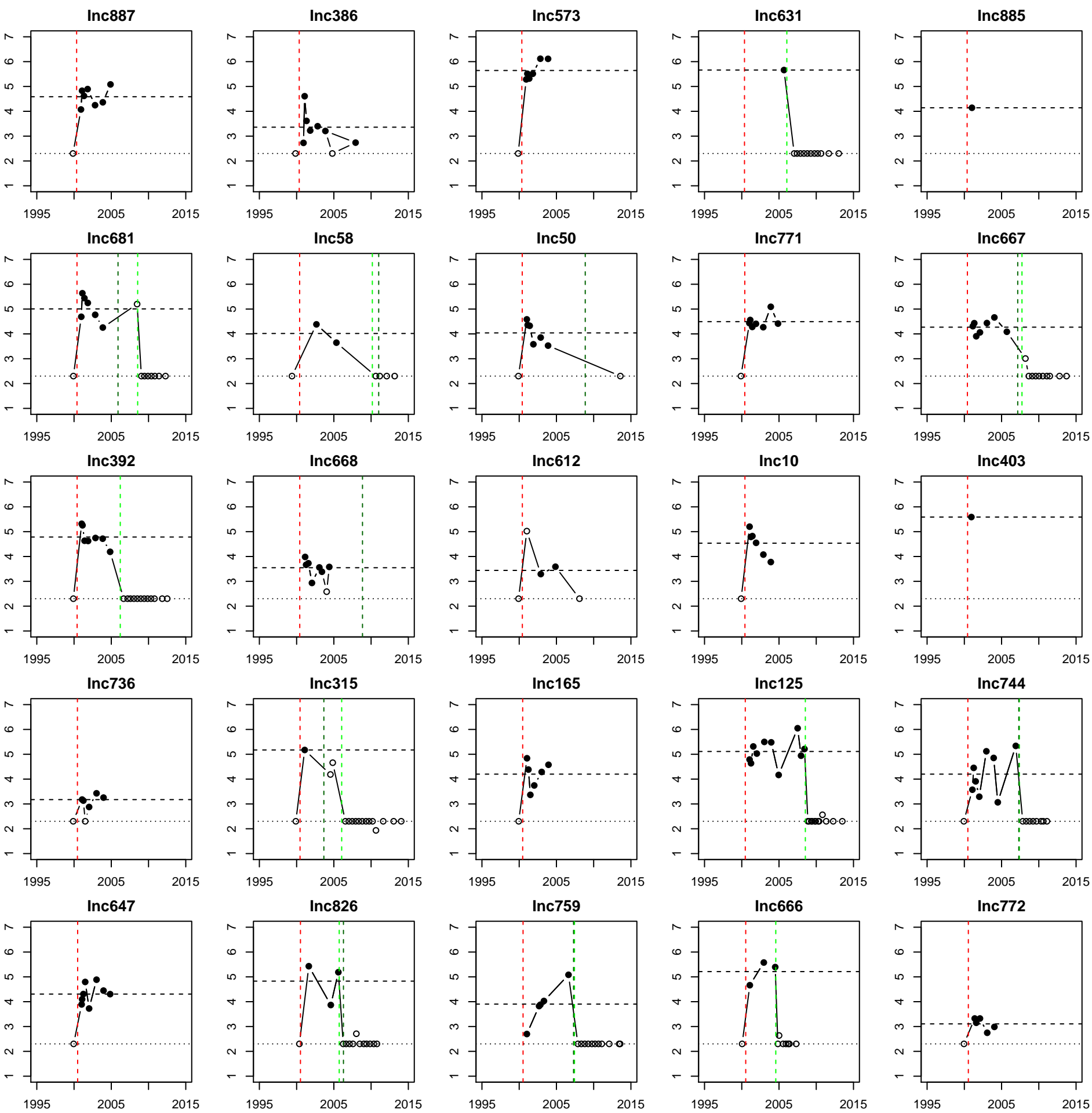

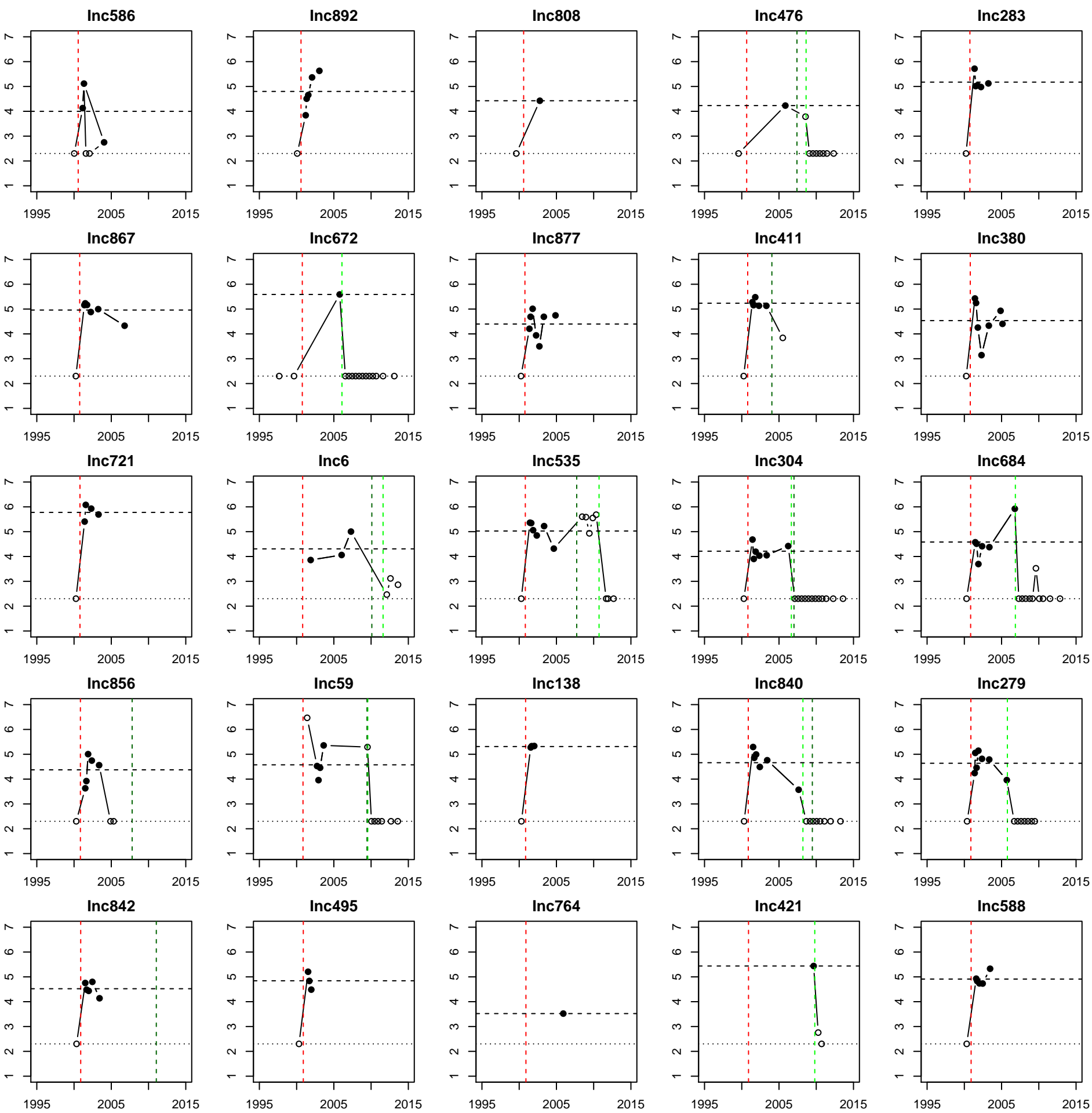

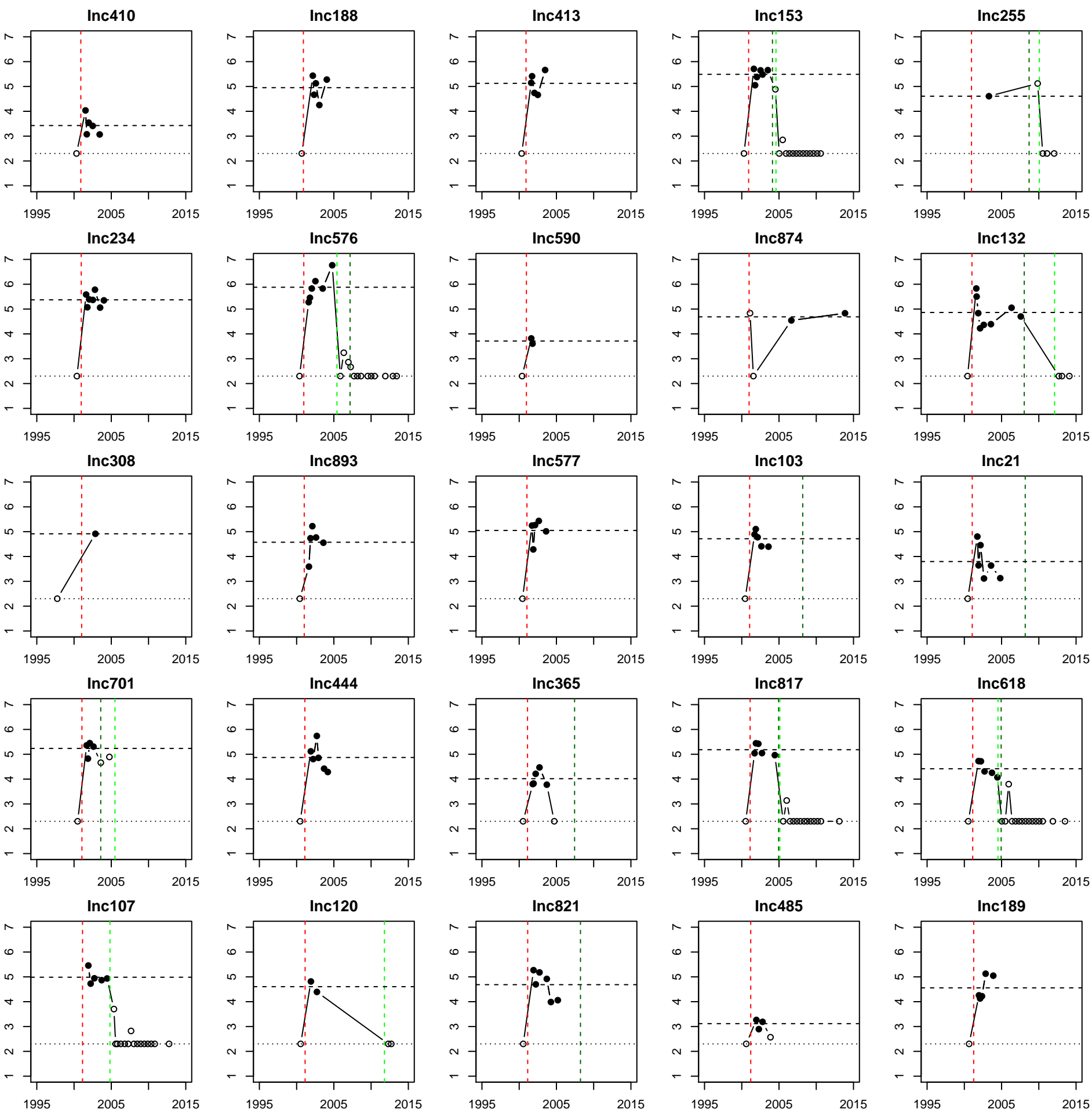

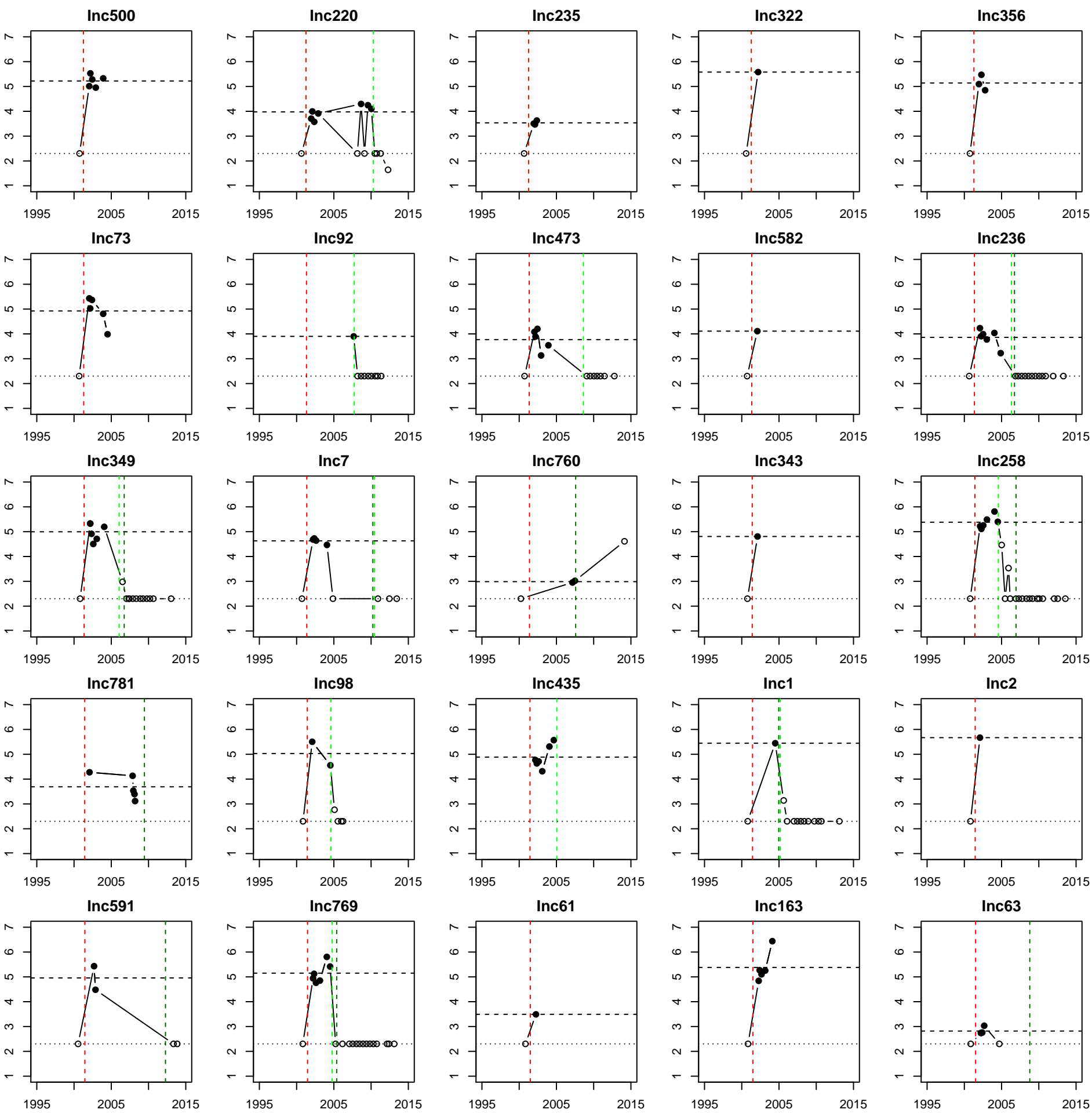

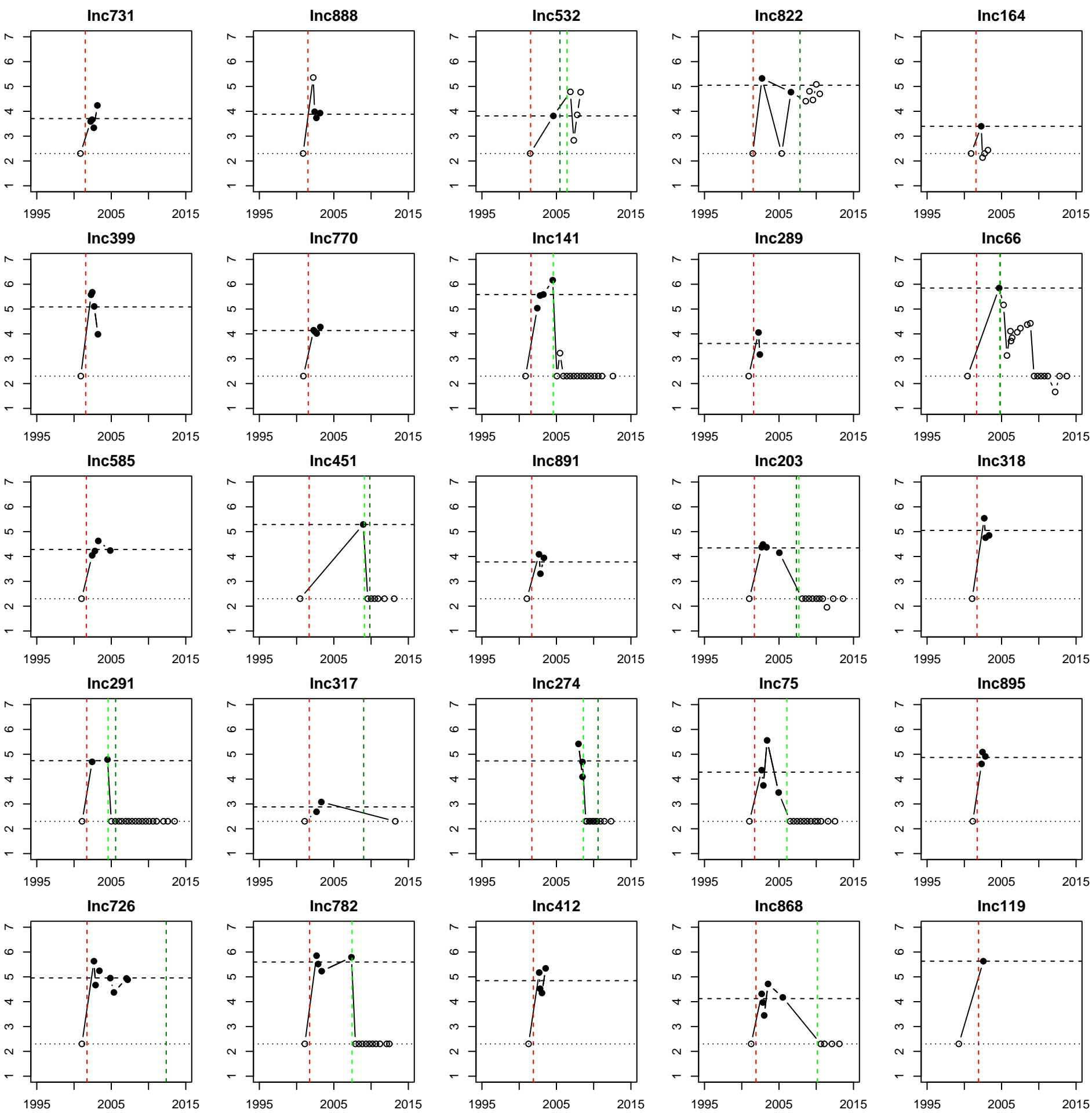

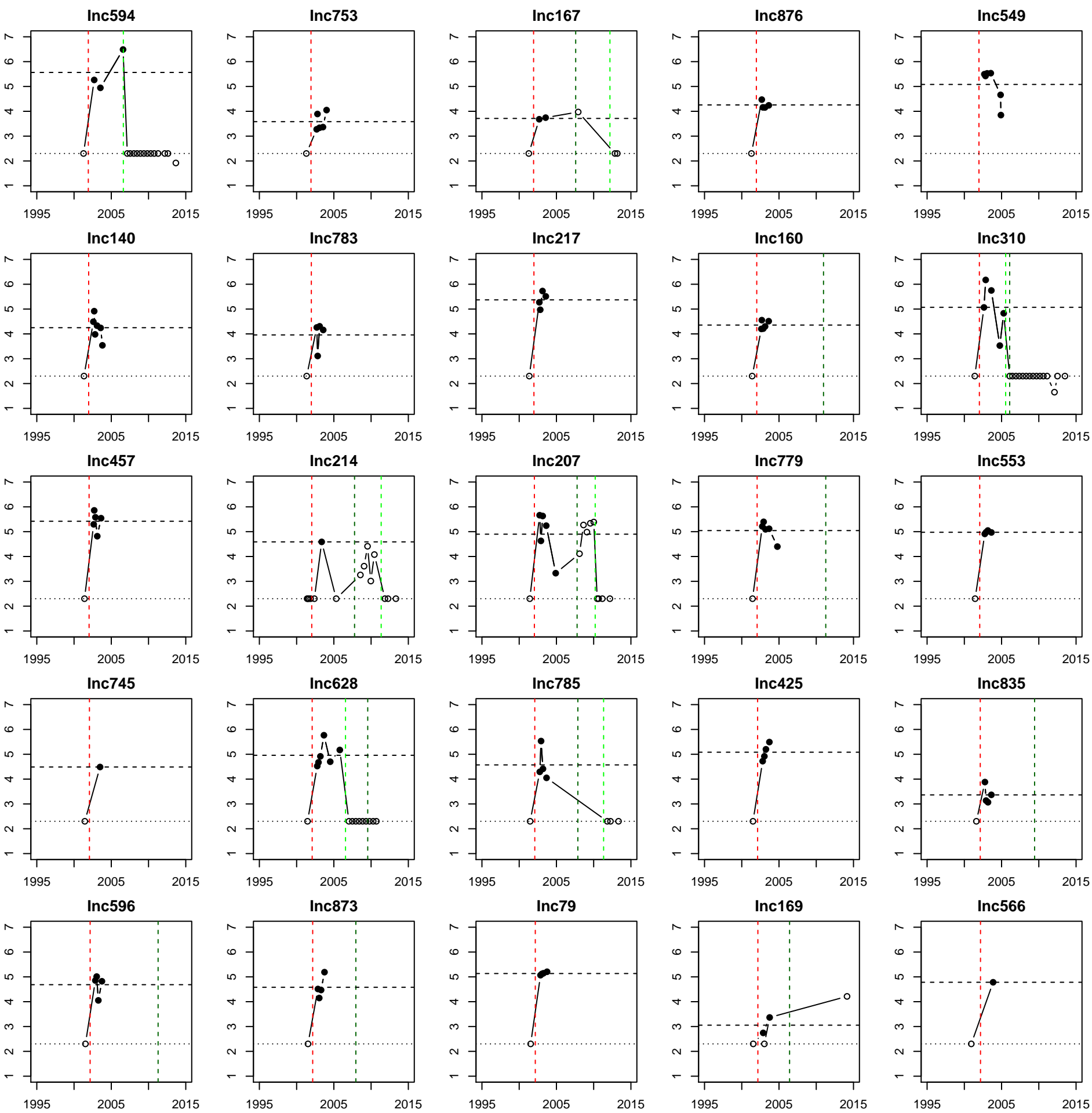

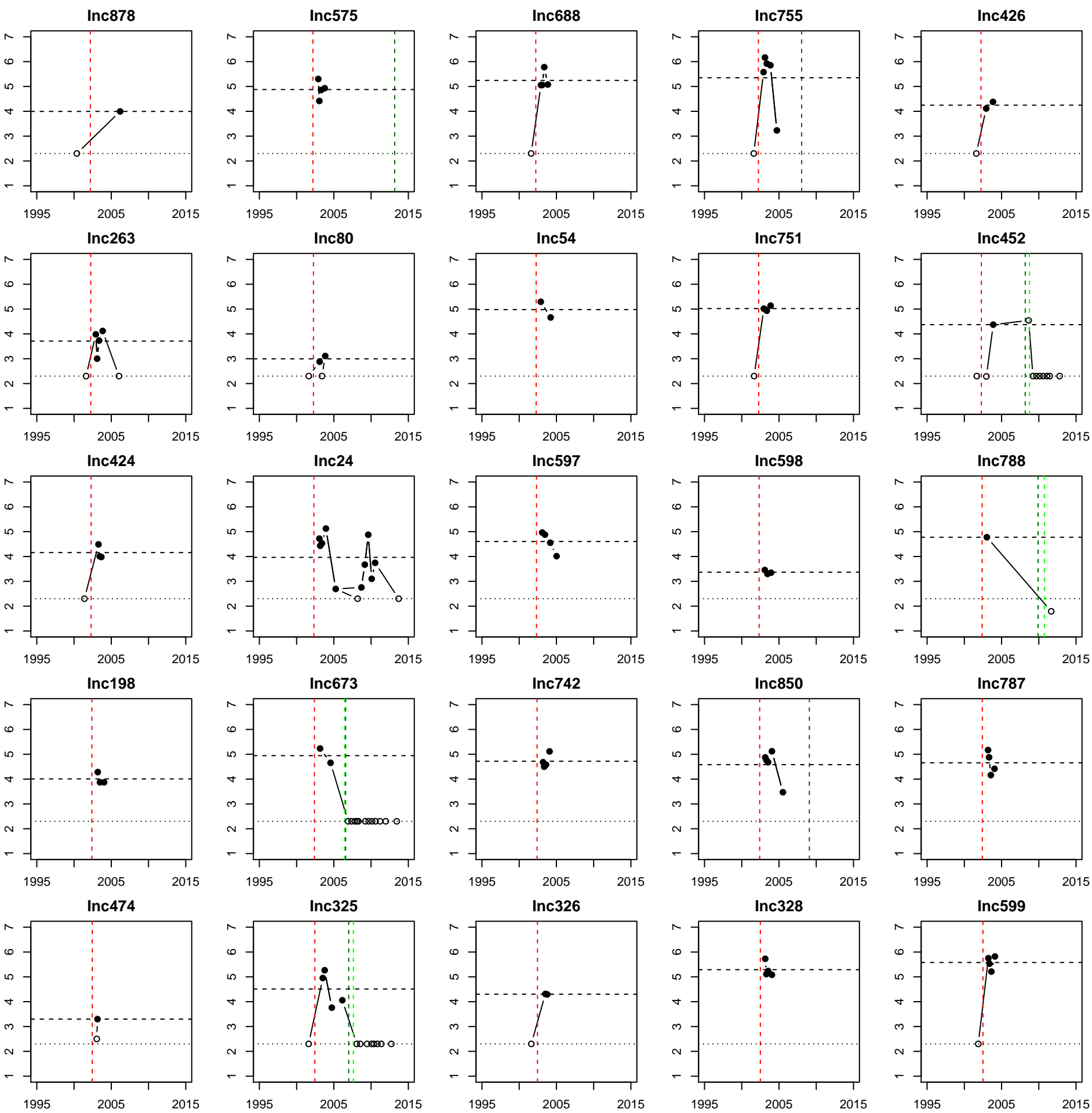

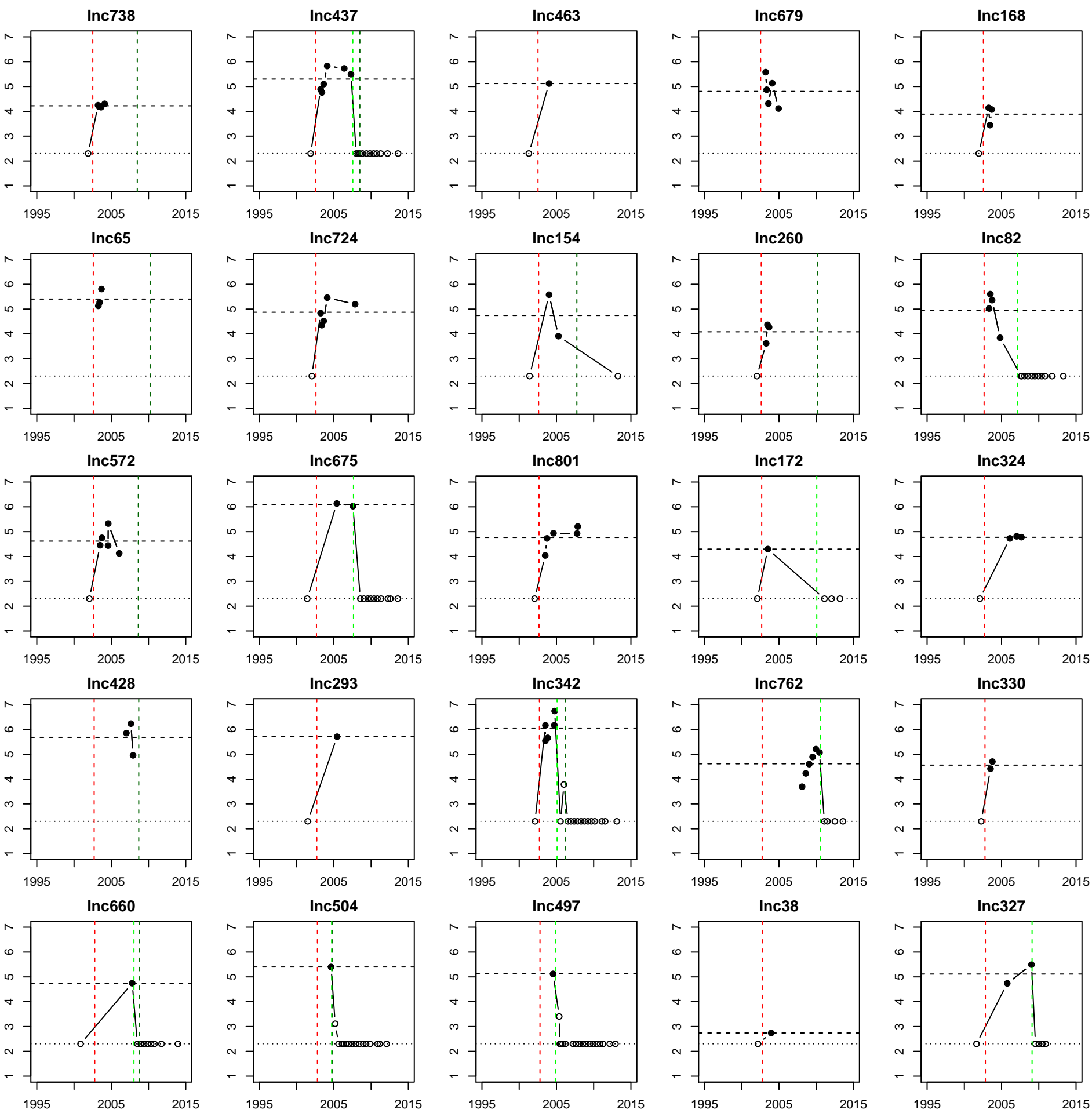

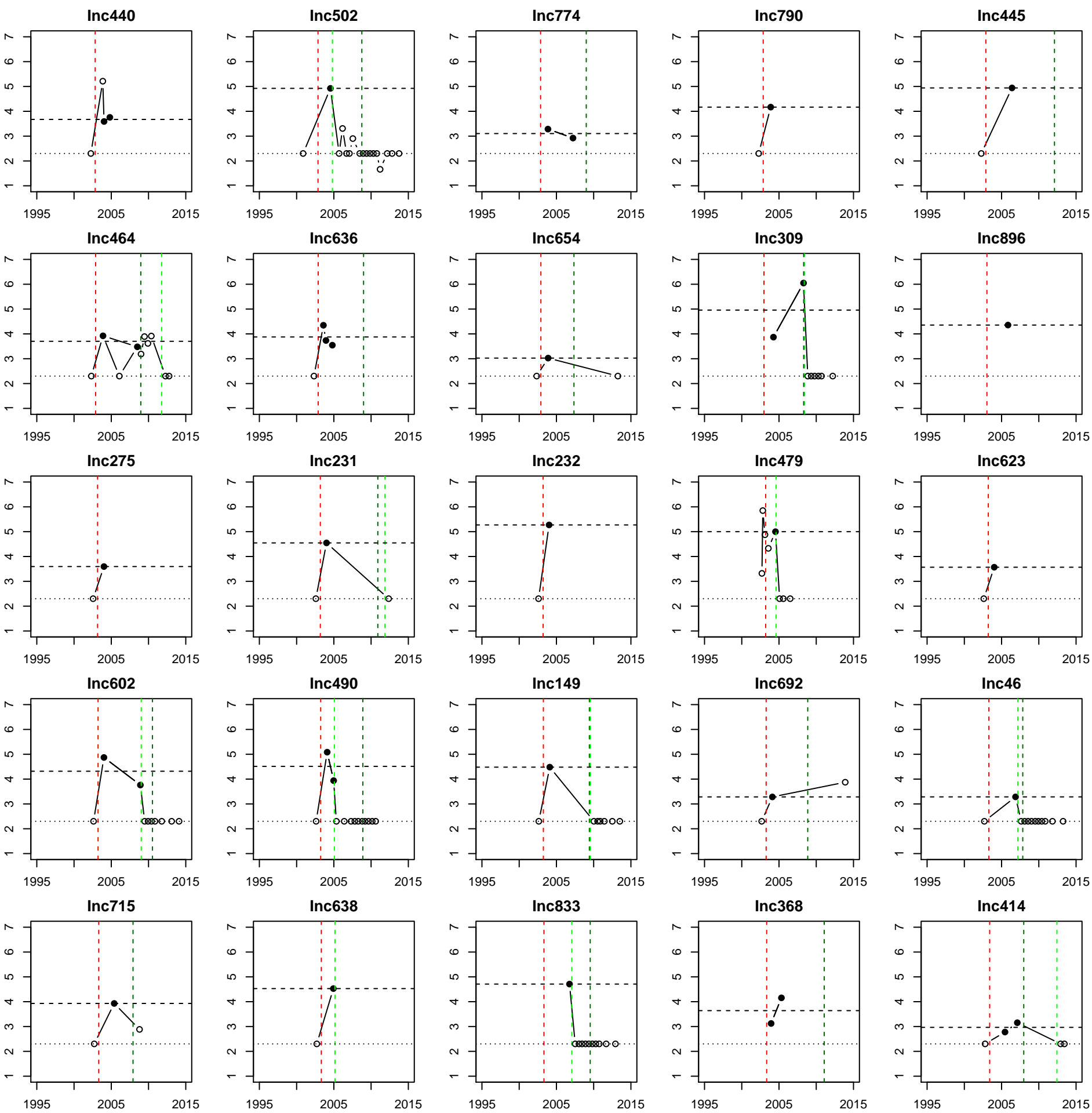

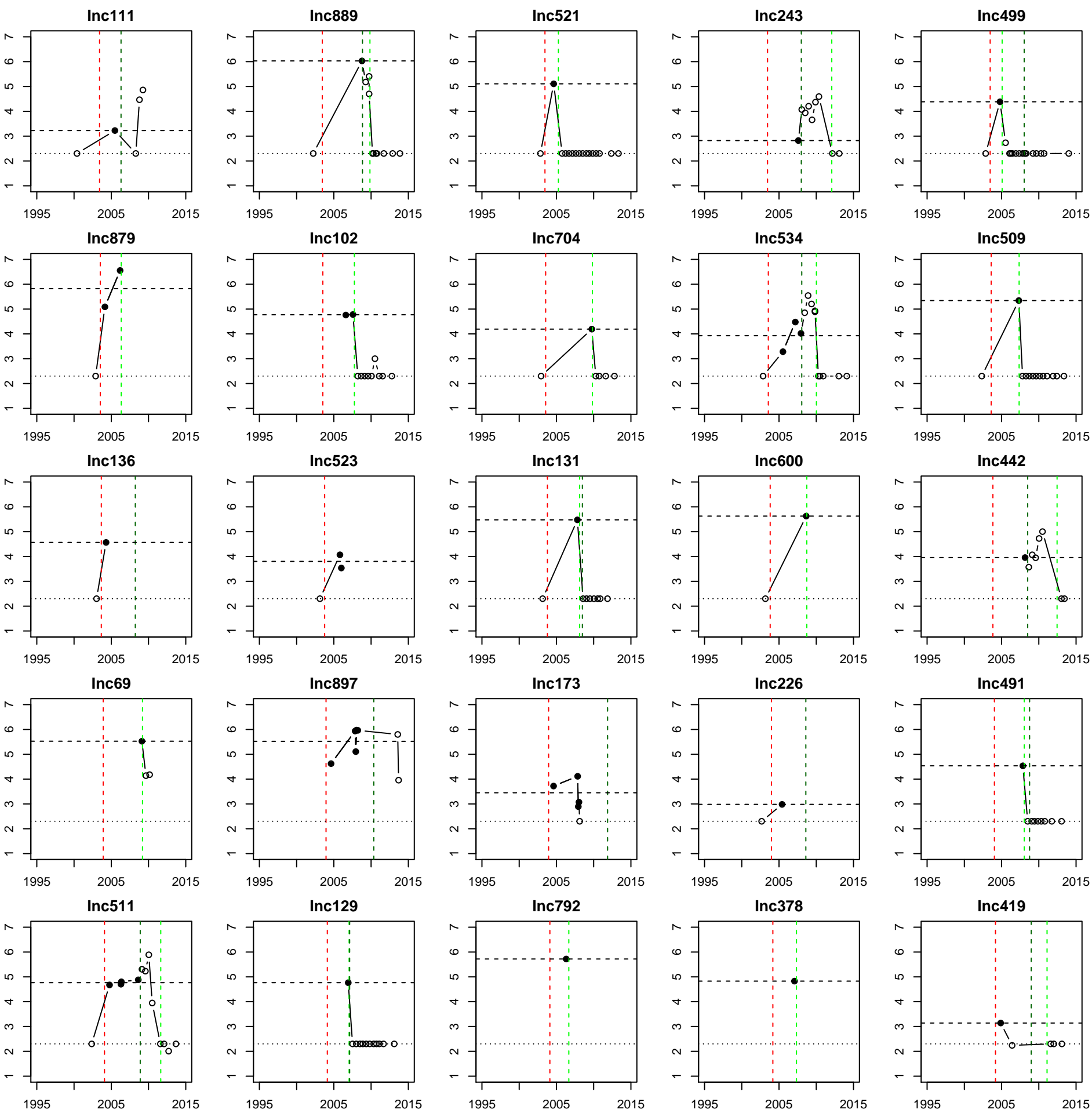

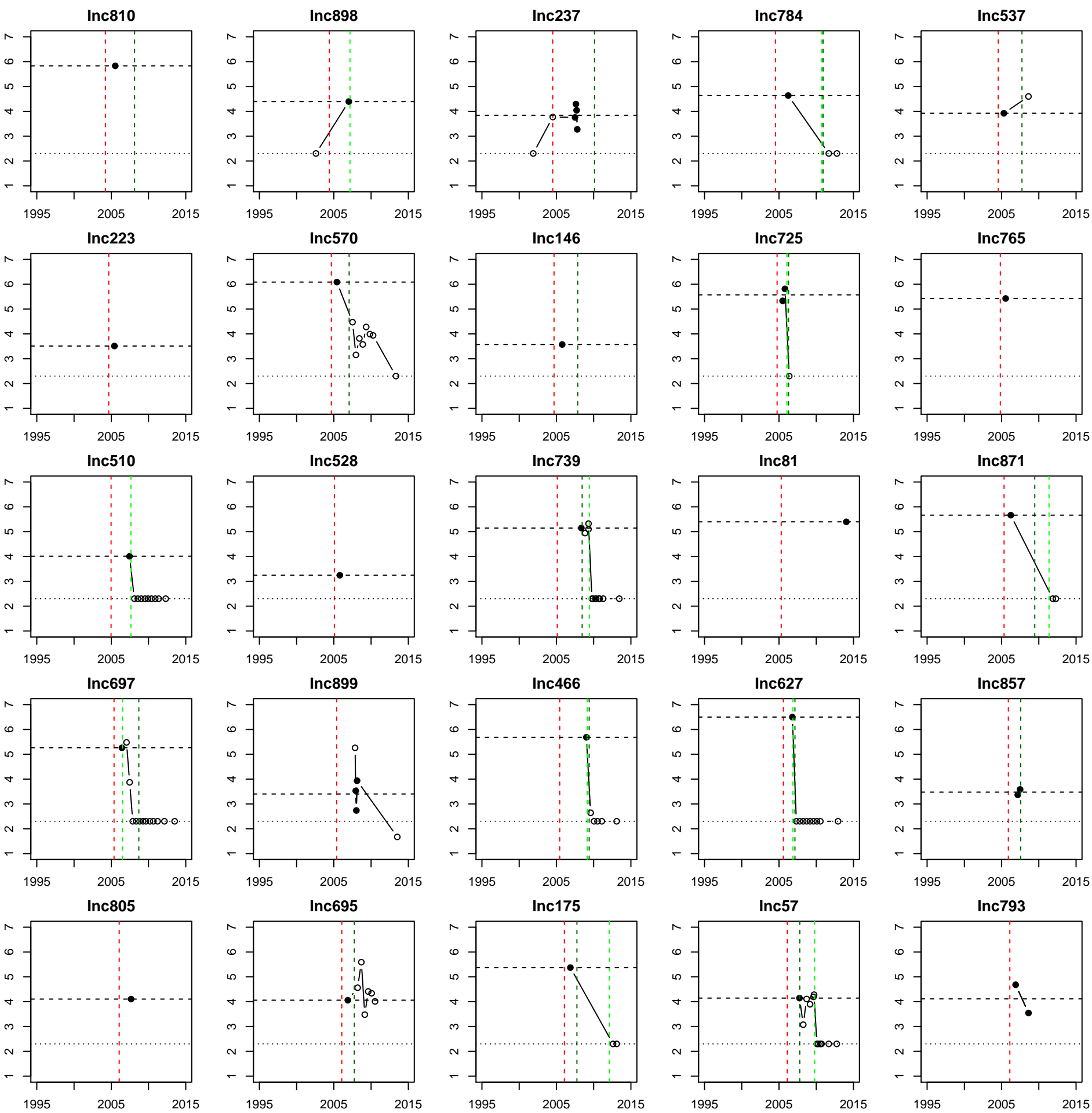

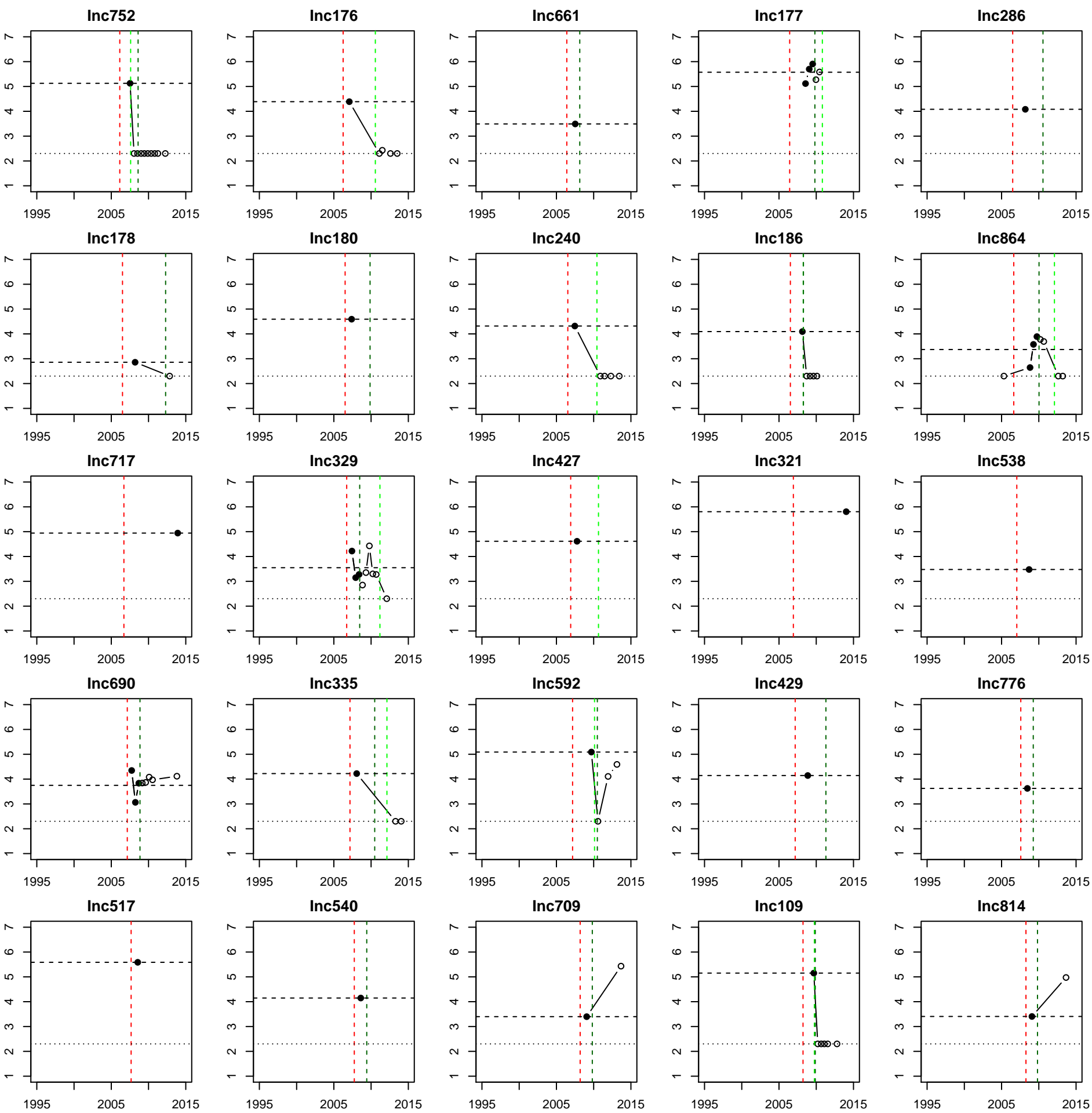

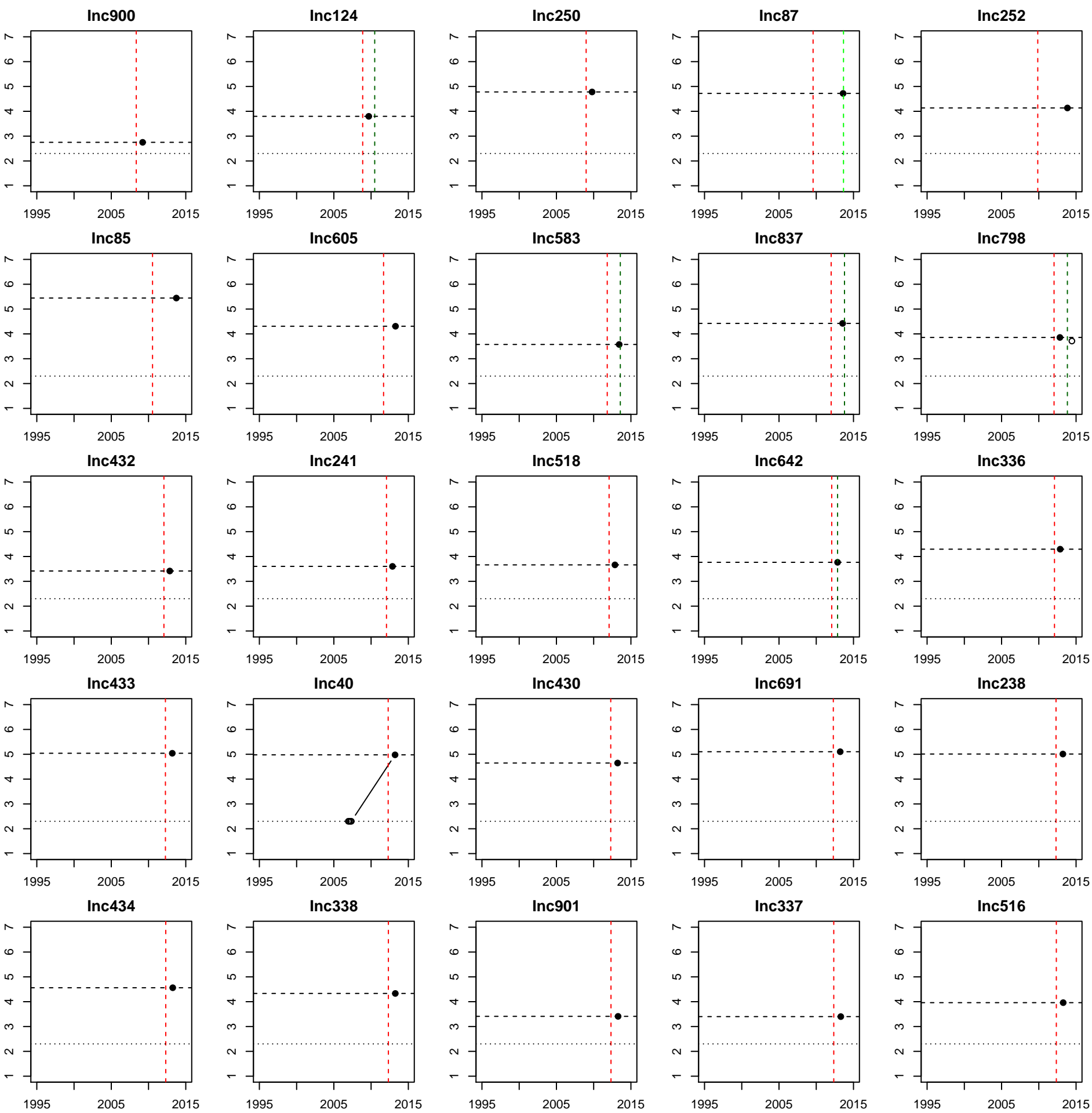

**Inc797**

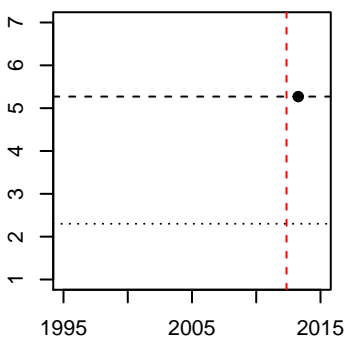

**Inc727**

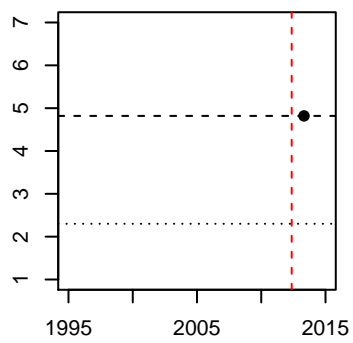

**Inc339**

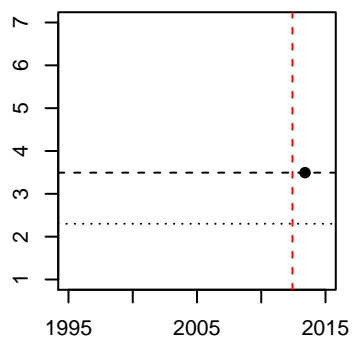

Supplement: Supplementary file 1. — Points are viral load values, shown as solid bullets when used for the SPVL calculation, and open circles otherwise. The vertical red line is the mid-point between last negative test and first positive test. The vertical light green line is the date ART started. The vertical dark green line is the date of first self-reported ART. The horizontal black line is the SPVL value. This data relates to Figure 2. DOI: http://dx.doi.org/10.7554/eLife.20492.018 [file elife-20492-supp1.pdf]
