# Supplementary material for: A transmission-virulence evolutionary trade-off explains attenuation of HIV-1 in Uganda
Source: eLife. 2016 Nov 5;5:e20492. doi: 10.7554/eLife.20492 (PMC5115872; doi:10.7554/eLife.20492)
Supplement: Reporting standard 1. — DOI: http://dx.doi.org/10.7554/eLife.20492.019 [file elife-20492-repstand1.pdf]

Analysis	Statistical Method	Sample size	Results	Where in the text
Relationship between transmission rate and SPVL	Maximum likelihood estimation of parameters and comparison of models based on Akaike Information Criterion (AIC).	n = 817	Model where transmission increases with three plateaus is favoured, ΔAIC = -75.96 compared to null model with a fixed transmission rate	Data file for figure 1<br>Line 93-115<br>Line 365-386
Relationship between time to AIDS and SPVL	Maximum likelihood estimation of parameters and comparison of models based on AIC.	n = 562<br>(incident cases with a SPVL value and information on time to AIDS)	Model where time to AIDS decreases with three plateaus is favoured, ΔAIC = 137.22 compared to null model with a fixed time to AIDS	Data file for figure 1<br>Line 116-134<br>Line 388-407
Temporal trends in SPVL	Multivariate linear regression of SPVL on date seroconversion and other covariates. Significance was assessed using type II analysis of variance. Confidence intervals (CI) determined assuming asymptotic normality of coefficients.	n = 603<br>(incident cases with a SPVL value)<br>n = 603	Adjusted for laboratory, assay, "visit" status, gender, age, subtype.<br>Change -0.033 log_{10} copies/mL per year<br>CI [-0.057; -0.009], p = 0.007	Line 525
			Adjusted for "visit" status, gender, age, subtype.<br>Change -0.022 log_{10} copies/mL per year<br>CI [-0.04; -0.002], p = 0.027<br><u>Effect of other covariates:</u><br>Males relative to females: +0.259 log_{10} viral copies/mL, CI [0.14; 0.38], $p = 4.2\ 10^{-5}$<br>Subtype D relative to A +0.211, CI [0.038; 0.38], p = 0.017<br>Age + 0.009 per year, CI [0.0008; 0.016], p = 0.030<br>"Visit" status -0.3 log_{10} copies/mL, CI [-0.51; -0.09], p = 0.006	Line 208-212<br>Line 526-527<br>Line 533<br>Line 534<br>Line 536<br>Supplementary file 1A, line 975-980
	Univariate linear regression of SPVL on date seroconversion. Significance and CI assessed as previously.	n = 603	Change -0.029 log_{10} copies/mL per year<br>CI [-0.045; -0.013], p = 0.0005	Line 218-219
Temporal trends in SPVL, including only viral loads sampled before 2004 (before ART was introduced)	Multivariate linear regression of SPVL on date seroconversion and other covariates. Significance and CI assessed as previously.	n = 442	Adjusted for gender, age, subtype<br>Change -0.019 log_{10} copies/mL per year<br>CI [-0.052; 0.014], p = 0.26	Line 249-253
Comparison of continuous versus categorical temporal trends	AIC comparison.	n = 603	ΔAIC = 7.2	Line 529
Test of normality of SPVL distribution	Shapiro-Wilk test.	n = 603	Rejection, p = 0.0018	Supplementary file 1A, line 966-968
Determinants of "undetectable" viral loads	Generalized linear model for "detectable" versus "undetectable". Significance was assessed using type II analysis of deviance.	n = 647<br>(all incident cases)	Supplementary file 1B	Supplementary file 1A, line 969-973<br>Supplementary file 1B
Effect of number of viral load measures on SPVL	Linear regression. Significance assessed using analysis of variance.	n = 603	No effect, $p = 0.61$	Supplementary file 1A, line 930
Temporal trends in subtype frequencies	Generalized linear model with multinomial response, for frequencies of the three subtypes as a function of the date of seroconversion. Significance assessed with analysis of deviance. CI determined by bootstrapping.	n = 551<br>incident cases with subtype A, D or R	Significance of date effect p = 0.044<br>Subtype A: 0.009 per year, bootstrap CI [-0.0007; 0.022]<br>Recombinants: 0.007 per year, CI [-0.005; 0.017]<br>Subtype D: -0.016, CI [-0.027; -0.002]	Line 276-282
